# Exploring recruitment strategies for place-based research in rural areas of Australia: a comparative case study analysis

**DOI:** 10.1186/s12875-025-03055-x

**Published:** 2025-11-26

**Authors:** Tracy L. Schumacher, Anna K. Jansson, Lucy Kocanda, Jennifer May, Leanne J. Brown, Clare E. Collins

**Affiliations:** 1 Department of Rural Health, University of Newcastle, Tamworth, NSW 2340 Australia; 2 Food and Nutrition Research Program, Hunter Medical Research Institute, New Lambton Heights, NSW 2305 Australia; 3 School of Health Sciences, College of Health, Medicine and Wellbeing, The University of Newcastle, Callaghan, NSW 2308 Australia

**Keywords:** Case study, Rural and remote, Recruitment, Heart health, Cardiovascular disease, Nutrition

## Abstract

**Background:**

Recruitment of participants into research studies is commonly challenging and becomes more difficult when targeting underrepresented population groups. Recruiting individuals living in rural locations, must use appropriate strategies if the sample size is to be achieved. The aim of this case study was to explore factors associated with research capacity, and to determine their impact on recruitment strategies, including timeframe and participant recruitment outcomes.

**Methods:**

This case study investigated the recruitment outcomes of four rurally based research projects performed between 2016-2024. Participants were enrolled either directly by the researchers (face-to-face method [f2f]), or through General Practitioners as trusted local intermediaries (TLI) through which all recruiting materials were passed. Projects were also categorised as large or small, according to the geographical size of the recruiting region. Cooke’s framework on measuring progress with research capacity building was used to create the dataset, with time and personnel used as the units of analysis. Data was analysed using cross-case synthesis and descriptive statistics.

**Results:**

When the researchers performed their own f2f recruitment, the smaller study delivered 132% of anticipated 50 health checks (n=66) and achieved 72% of 25 interviews (n=18) across three separate time-points. The larger f2f study recruited 674 participants over seven events. The smaller TLI study took twice as long to recruit participants than planned (4 years) and achieved n=25 (25%) of the sample size. The larger TLI study took 43% longer (4.3 years) and achieved 44% (n=132) of the target sample size. Key factors in research capacity building were linked to staffing continuity and sustainability, as well as partnerships and collaborations that impact on recruitment. High turnover of staff affected both TLI studies, with one having eight different people in the recruitment role over two years. All studies were adapted iteratively to ensure the research was ‘close to practice’.

**Conclusion:**

Recruitment in rural settings is complex with many factors impacting recruitment success. Researchers had greater capacity to recruit participants f2f, using intensive periods which was less affected by staff turnover. Both the research focus and recruitment strategy need to be ‘close to practice’ for all involved.

**Trial registration:**

RuralCVD: n/a, CHAaRGE: 20: n/a, Health SMaRT: n/a, Healthy Rural Hearts: ACTRN12621001495819 (Date registered: 03/11/2021).

**Supplementary Information:**

The online version contains supplementary material available at 10.1186/s12875-025-03055-x.

## Background

Participant recruitment is a practical challenge for many research studies, as well as an activity with significant time and cost impacts. Despite the high importance, recruiting methodology is rarely described with the same level of detail as other methodological components within published research studies. Strategies are required to be nuanced and targeted if the study is to enrol a sample that is representative of the population for which the intervention is intended and of the required sample size [1]. People who live in rural locations are an example of a population group under-represented in health research, and where definitions of “health” and health behaviours vary [2, 3].

Rural populations experience considerably higher rates of health disparities compared to people living in metropolitan areas [4, 5]. These health disparities are not due to rurality itself, but linked to unique challenges associated with rurality. These include, but are not limited to access to healthcare services, health workforce shortages, socioeconomic disparities, and an ageing population [4, 6–8]. Recruitment of rural populations into health research requires accounting for these challenges, and yet remains necessary to increase health equity. Reviews of studies recruiting under-represented populations, which include rural people, have shown socioeconomic and cultural factors, awareness and opportunity barriers, mistrust and potential healthcare provider biases to be barriers [9, 10]. Strategies previously used to overcome these barriers include face-to-face contact with prospective participants, advertising through media or letter, online platforms, or third-party assistance (e.g., trusted local intermediaries) [11, 12].

Collaborations between place-based experts and local leaders have also been recommended as a solution to overcome the health inequities faced by rural people [13]. Adapting research methodologies to account for being “place-based” is not a new phenomenon [14] and include proposed roles such as process facilitator or knowledge broker [15]. However, in the case of place-based experts recruiting rural population groups into research studies, it is problematic to assume a workforce could already be in place. In Australia, many barriers have been identified that challenge the sustainability of the rural workforce for healthcare and research [16], where job security, career progression, workload and the ability to develop and secure large scale grants have been identified as a hindrance. This extends to clinicians doing research, who have found rural research to be fragmented, poorly resourced and needed an underlying system of support [17].

### Case study background

The case study below describes the experiences of place-based researchers when recruiting into four different studies, each with a focus on nutrition-related health behaviours. Dietary intake is recognised as a modifiable lifestyle risk factor that impacts on the development of chronic disease [18]. Modelled risk factors using Australian data indicate that if people living outside of metropolitan areas experienced the same level of lifestyle-based risk factors as those living in major cities, 11% of cardiovascular disease deaths would be delayed or averted [19]. Yet, there have been few rurally targeted interventions [20] and limited funding invested in nutrition research delivered specifically in rural and remote communities in Australia [21].

Two different methods were used to recruit participants into the studies. The first involved the researchers proactively recruiting participants themselves, referred to hereafter as face-to-face (f2f). The second reactively recruited, using trusted local intermediaries (TLIs) in the form of primary health care professionals, specifically general practitioners (GPs) and the staff associated with the practice [12]. These two methods may differ in the type of demographic characteristics within the population recruited, such as education level or income [12]. Face-to-face recruitment can include members of the research team attending community events and promoting their research using verbal scripts and recruitment flyers. While it provides community members an opportunity to directly meet members of the research team, it can be a resource-intensive task. Doctors are commonly identified as a TLI, and while they play an important role in facilitating patient’s decision-making and attitudes towards enrolling in research trials, factors such as the clinician’s attitude towards the research, lack of recognition for referring patients and lack of financial incentives can influence their willingness for helping recruit to research [22, 23].

Given the limited published rural research and lack of knowledge of successful recruitment for health-promoting research in rural and remote areas in Australia, the aim of this case study was to explore two different participant recruitment methods used by a place-based Australian rural research team, across four separate studies between 2016 and 2024. In particular, we aimed to explore factors associated with research capacity, and to determine their effect on recruitment timeframes and participant recruitment outcomes.

## Method

### Study design

This case study was guided by methods described by Yin [24]; (i) case study questions, (ii) propositions, (iii) units of analysis, (iv) how data are linked to the propositions, and (v) how the findings were interpreted. It used a cross-case synthesis with pattern matching to explore the effect research capacity when using f2f and TLI as proactive and reactive recruitment methods, in small and large recruiting regions, and how these influenced recruitment outcomes when engaging rural people in primary health care research. This case study used time and personnel as the units of analysis. These units of analysis were chosen as they are integral to research proposals, timelines, and budgets.

Data were included from four separate research trials conducted between 2016 and 2024. The four research trials had four commonalities: (1) the research group was the same; (2) the target population were adults who lived in rural areas of New South Wales, Australia; (3) all trials were based on the COM-B theory of change [25] and; (4) the overarching goal of all trials was to engage the target population with their health, and their healthcare team, with a focus on nutrition. All studies were approved by the University of Newcastle Human Research Ethics Committee. The four studies were:

1. RuralCVD, a pilot randomised controlled trial that used provided medical nutrition therapy via telehealth for the prevention of heart disease, targeting people in a single rural town. This study intended to follow participants for three months and ran from November 2015- January 2019. Eligibility criteria were based on current health status, as assessed by GP. Ethics approval number: H-2015-0427.
2. **C**hanging Health Actions at Rural and regional Events in 20 minutes (CHAaRGE:20) [26, 27], a cross-sectional study designed to deliver a brief nutrition-related health intervention to people at large scale events in the area, within a relatively short period of time. This study ran from August 2017-August 2022. Eligibility criteria were adults attending event. Ethics approval number: H-2017-0197.
3. Health Services, Medicine and Rural Towns (Health SMaRT) [28] was designed as a small cross-sectional study to determine which health measures related to the prevention of heart disease were of particular interest to populations who did not regularly engage with primary health care services. This study was conducted between February 2018 – October 2018. Eligibility criteria were adults attending event. Ethics approval number: H-2018-0005.
4. Healthy Rural Hearts (HealthyRHearts) [29, 30] was a randomised controlled trial that covered a large rural geographic area and aimed to provide medical nutrition therapy via telehealth for the primary and secondary prevention of heart disease. This study intended a 12-month follow-up period. This study was conducted between September 2020 – October 2024. Eligibility criteria was based on current health status, as assessed by GP. Ethics approval number: H-2021-0193.

Health SMaRT and CHAaRGE:20, with a health promotion focus, relied on researchers to recruit study participants themselves, using the f2f method. RuralCVD and HealthyRHearts, with a more specific cardiovascular focus, recruited using general practices as a trusted local intermediary to recruit participants to the study, and to whom all recruiting material was distributed. Each study was additionally categorised according to the size of the recruiting area: small and large. Small areas were defined as sites where the potential pool of participants was limited. Large areas were considered to have much greater access to potential participants. They are defined here as a geographical area encompassing a health district and events with attendee estimates of 30,000–50,000. A summary of the commonalities, recruitment methods and study recruitment sizes can be seen in Supplementary Fig. 1: Overview of case study design.

### Case study setting

Recruiting for the four studies were primarily led by nutrition and dietetics researchers from the University of Newcastle Department of Rural Health (UONDRH). The UONDRH was established in 2001 with the aim to “…*improve health and wellbeing of people in regional*,* rural and Indigenous communities”.* The UONDRH is one of 19 Departments of Rural Health and is funded by the Australian Government Department of Health under the Rural Health Multidisciplinary training program [31, 32]. The UONDRH covers a land area of approximately 143,120 km^2^ (approx. 18% of New South Wales [NSW]), including small to large rural towns [33, 34]. While the UONDRH has been established for over 20 years, its researchers have limited experience with recruitment of rural people for health-related research trials [35].

The New England North-West region is the geographical setting for these studies, and part of the area served by UONDRH. Compared to the rest of Australia, the area is inequitable on many demographic and health-related statistics e.g., median weekly household income ($1,328 versus $1,746), population tertiary educated (15% versus 30%) and having 1–3 long-term health conditions (48% versus 40%) [36]. While heart-related hospital admissions are similar to NSW (age standardised rate [ASR]: 40.4 versus 39.3 per 10,000 persons), and slightly below the national level (ASR: 42.3 per 10,000 persons), the New England and North-West area experience a considerable higher rate of coronary heart disease mortality (ASR: 85.0 per 10,000 persons) compared to national (ASR: 63.8 per 10,000 persons) and NSW (ASR: 62.4 per 10,000 persons) averages [37].

### Case study process

Efforts were made to identify theoretical propositions that could provide direction for the collection and analysis of data. A framework measuring research capacity building was developed based on work by Cooke [38], and used to create a data set for this case study (see Table 1). The framework includes the capacity of the immediate and extended (internal) research team and partner (external) organisations. Five of Cooke’s six principles have been used here as propositions [38]:

**Table 1:** Propositions based on the principles by Cooke (2005) [38], including case study data sources

Case study propositions	Sources of case study evidence
*Research capacity is built by developing appropriate skills and confidence*	
**Internal:** Planned capacity building that relates to building confidence and upskilling within the research team and accounts for their professional role, including the Chief Investigative team and personnel. *Examples may include* workshops and training delivered to project personnel, supervision/mentoring provided, material/resources developed.	• Ethics applications • Staff training protocols & staff handovers • Emails/phone records/email templates • Diary appointments • Study resources • External referrals • Interviews with research/CI team
**External:** Planned capacity building that relates to building confidence and upskilling external organisations and individuals involved in individual projects. *Examples may include* materials/resources developed for informational/skill development, mentoring/coaching provided, personnel responsibilities including provision of upskilling and confidence building, and those specific for professional roles.	
*Research capacity building should support research ‘close to practice’*	
**Internal:** Justification of research topics of importance to team and surrounding areas, as well as study design, processes and culture within the rural research team. *Examples may include* chief investigative and research team members experience, recruitment of rural personnel for research team and processes suitable for team and location.	• Recruiting materials and participant information statements • Training materials • Reports provided to organisations and participants • Recruitment notes and handovers. • Ethics applications and variations • Job descriptions and qualifications of personnel • Interviews with research/CI team
**External:** Study design and strategies planned to ensure the research is relevant to service users and local concerns. The recruitment plans are appropriate for the users. *Examples may include* assessed interest to participate, planned and changes to recruitment strategies/processes, recruitment outcomes, personnel recruiting at public events.	
*Linkages*,* partnerships and collaborations enhance research capacity building*	
**Internal:** Planned development of new or existing linkages, partnerships or collaborations with internal sources for recruitment purposes. *Examples may include* cross-disciplinary research team, or connections within wider organisation at other campuses.	• Email/phone records • Study protocols and ethics applications • Interviews with research/CI team
**External:** Planned capacity building to enhance recruitment through supporting linkages, partnerships and collaborations with external organisations, special interest and volunteer groups, and prospective members of the public. *Examples may include* strategies used to develop and support partnership with general practices and their staff.	
*Research capacity building should include elements of continuity and sustainability*	
**Internal:** Include any strategies designed to address continuity and sustainability of internal research staff, including those specific to individual studies. *Examples may include* training staff in tasks that may be used on other studies or engaging junior staff for development.	• Study protocols • Training manuals • Ethics applications and variations • Email records • Interviews with research/CI team
**External:** Include any strategies designed to engage and retain external organisations and members of the public in the research studies, including strategies for follow-up completion, and engagement in future projects with rural researchers again. *Examples may include* strategies or resources used to enhance longevity of engagement with external parties.	
*Appropriate infrastructures enhance research capacity building*	
**Internal:** Include the purchase/use of equipment, space and technologies required for the completion of the study *Examples may include* office equipment, communication technology, secure transmission and storage of data, as well as equipment specific to the project.	• Email/phone records • Service agreements • Records from Study and safety protocols and ethics applications describing the use of equipment and technology • Interviews with research/CI team
**External:** Include any equipment or technology required for the use of personnel external to the research team. *Examples may include* communication technology, such as referral software or secure videoconferencing.	

*Internal relates to capacity building of the immediate and extended research team*,* and external relates to capacity building of people belonging to partner organisations*,* intermediaries*,* and other rural populations*

1. Research capacity is built by developing appropriate skills and confidence, through training and creating opportunities to apply skills. This includes opportunities and activities with the primary aim of building skills and confidence.
2. Research capacity building should support research ‘close to practice’ in order for it to be useful. This includes research that is immediately useful and has direct relevance to decision making of participants, intermediaries, and the research team.
3. Linkages, partnerships, and collaborations enhance research capacity building. This includes practice knowledge that is shared and developed between participants, intermediaries, and the research team.
4. Research capacity building should include elements of continuity and sustainability. This includes opportunities to practice newly acquired skills and the sustainability of personnel.
5. Appropriate infrastructures enhance research capacity building. Infrastructure is defined here as access to equipment and services that are required for the research projects to be conducted.

### Data sources

A database of evidence to support the case study was established, using data obtained from documents and records (e.g., journal publications, ethics submissions and grant outcomes), researcher records (e.g., unpublished data, staff records), local observations, interviews and focus groups with CI’s and research team members (where applicable for each trial, see Supplementary Material 1: Interview guide). The data relating to the units of analysis (personnel and time) and the outcomes of interest included project funding (i.e., length of funding, AUD$ and source), personnel (staff, student, volunteer etc.), participant characteristics, recruitment strategies/processes and number of participants/practices recruited and retained. Outcomes were compared between recruiting methods i.e., trusted local intermediaries (TLI) and researchers face to face (f2f).

### Data terminology

The included studies were dichotomised based on recruitment method, namely TLI and f2f. Time is described in either years, months, weeks, or days. The internal research team is dichotomised into the immediate (or local members) and the extended (or metropolitan-based). Staffing was described as full-time equivalents (FTE), where 0.2 FTE represents one day per week and 1.0 FTE represent full-time. Staff were described as chief investigator (CI), early career researcher (ECR), research officers (RO), research assistant (RA), Doctor of Philosophy candidate (PhD candidate) or undergraduate student volunteers. Funding obtained for the research studies were categorised as internal, external or in-kind contributions. Internal funding was granted by the University of Newcastle, whereas external funding was granted by competitive external government or non-government organisations. In-kind contributions were resources that were used by the recruiting team (e.g. time, personnel, equipment), and that were consistent with organisation’s purpose or mission.

### Data analysis and interpretation

Data were analysed using a cross-case synthesis, comparing TLI and f2f as recruiting methods in two studies with small recruiting areas, and two studies covering populations over an extended area. Findings were aggregated across the studies and synthesised according to study propositions. To reduce investigator bias, a researcher not part of any of the studies (AKJ) extracted data from the records and performed the analysis and interpretation.

## Results

An overview of the included studies, including their allocated and required time and funding are presented in Table 2.

**Table 2:** Overview of included studies

Study	Recruiting site size	Design	Study aim(s)	Time and funding ($AUD)		Sample size	
				Allocated	Required	Target	Achieved
**Studies using Trusted Local Intermediaries (TLI)**							
RuralCVD	Small: Single rural town (50K population)	Pilot RCT	Translate in-person dietary intervention program for CVD prevention to a telephone consult. Measure 3-month heart disease risk biomarkers, diet and QoL. Evaluate the feasibility and acceptability.	Total allocated time: 2 years Intervention length: 5 weeks Follow up length: 3 months Allocated staffing: RA1: 0.2FTE – 10 months Dietitian: 0.2FTE – 14 months Proposed funding for project: $30,096 (Funding covered FTE & pathology)	Total required time: 4 years Intervention length: 5 weeks Follow up length: 3 months Required staffing: RA1: 0.2FTE −10 months RA2: 1.0FTE - 2 weeks Dietitian: 0.2FTE – 14 months ECR: 0.2FTE – 7 months PhD candidate: thesis time Total funding for project: $50,441 + in-kind (Additional funding covered FTE and qualitative evaluation)	*N*=100 screened for eligibility *N*=35 complete study	*N*=3 general practices consented *N*=25 participants screened *N*=8 participants completed study
HealthyRHearts	Large: Large geographic area in NW NSW	RCT	To test feasibility, acceptability and cost-effectiveness of a Medical Nutrition Therapy primary and secondary CVD prevention program delivered by telehealth that meets needs of GPs, primary care staff, dietitian and patients in regional and rural areas. *Primary outcome*: total serum cholesterol	Total allocated time: 3 years Intervention length: 6 months Follow up length: 12 months Allocated FTE: Recruiters: 1.3FTE (3 years) Recruiter (in-kind): 0.05FTE (1 year) E/MCR: 0.8FTE (3 years) Proposed funding for project: $1,028,236.00 (Funding covered FTE, pathology and associated study costs)	Total required time: 4.3 years Intervention length: 6 months Follow up length: 12 months Required FTE: Recruiters: 1.25 FTE (Year 2) + In-kind: 0.05 (6 months) 0.8 FTE (Year 3) 0.4 FTE (Year 4) E/MCR: 0.4FTE (5 years) Total funding for project: $1,028,236.00 + in-kind	*N* = 30 GP practices with 10 eligible participants per practice *N* = 300 eligible participants	*N* = 24 general practices consented *N*= 15 practices actively recruited (12.8 participants per practice, range 4–38) *N*=192 participants recruited *N*=132 eligible participants *N*= 79 completed study as of 06/06/2024
**Studies using Face-to-face (f2f) methods**							
Health SMaRT	Small + large: Small rural town <3K population, One large regional event	Mixed methods	Trial three methods of engaging with CVD in rural people who may have low health literacy levels or low engagement with health professionals. Identify which recruitment strategies, materials and opportunities need to be in place for rural people with low technology confidence and low health literacy to make engaging with telehealth services as easy as possible. Identify barriers that health professionals offering telehealth services may face in planning and delivering services.	Total allocated time: 12 months Intervention length: 20-30minutes Follow up length: Follow-up interviews only Allocated FTE: In-kind: 2 x E/MCR at events Data collection + analysis: 5 days Interviews + analysis: 9 days Total funding for project: $9,834 + in-kind (Funding covered interview costs and consumables)	Total allocated time: 12 months Intervention length: 20- minutes Follow up length: n/a Required FTE: Data collection: 5 days In-kind: 2 x E/MCR at events PhD candidate: 0.4FTE (1 year) Interview analysis: 4 days Total funding for project: $7,792 + in-kind	*N* = 92 (max) 25 - 42 interviews 40 - 50 health checks	*N*=80 Health checks: *N*=66 *N*=3 from special populations *N*=63 from markets/AgQuip Interviews: *N*=18 *N*=0 from rural people via markets *N*=8 from rural people via promotion through networks *N*=10 health professionals
CHAaRGE:20	Large: Two annual regional events attracting >80K people	Repeated cross-sectional	To describe the current health status of participants attending rural-based festivals using opportunistic engagement in health-related activities. To identify areas for targeted brief health interventions suitable for opportunistic delivery at rural festival events.	Total allocated time: n/a Intervention length: 20 minutes Follow up length: n/a Allocated FTE: In-kind: 2 staff + 2–3 students per day per event Total funding for project: No external funding	Total allocated time: n/a Intervention length: 20 minutes Follow up length: n/a Allocated FTE: In-kind: 1–6 staff + 2–6 students per day per event Total funding for project: No external funding	*N*=Unknown Upper limit set at 10,000.	*N*=674

*E/MCR* Early/Mid-career researcher, *FTE* Full time equivalent, *GP* General Practitioner, *n/a* Not applicable, *NW NSW* Northwest New South Wales (Australia), *PhD* Postgraduate doctoral student, *RA* Research assistant, *RCT* Randomised Controlled Trial

* People from health checks were also able to provide interviews

### Time, funding and sample size outcomes

It took considerably longer to recruit into the research studies than initially anticipated for both studies using TLI. For RuralCVD, recruitment to took twice as long as initially planned (i.e., 4 years instead of 2 years). This exceeded the original allocated budget by 67%, in addition to in-kind support provided, due to the resources and personnel required to continue recruitment. Despite the additional time and budget commitment, the study only managed to screen 25% of the target sample size for eligibility screening (target *n* = 100) and achieved 23% of the target completion rate (completion target *n* = 35).

For HealthyRHearts, the second TLI study, no further external funds were allocated to the budget, but completion was prolonged by 1.3 years, (43% longer), with follow-up continuing until October 2024. The study recruited 80% of the target number of GP practices and 44% of the target number of participants. Of the 126 general practices listed as eligible in the area, 7% (*n* = 9) were no longer operating, 19% (*n* = 24) provided practice consent and 74% (*n* = 93) declined to participate. It must be noted that this study was recruiting during the COVID pandemic, when rates of COVID infections were increasing and many of the local general practices were focused on providing immunisations to the population.

CHAaRGE:20 commenced in 2017 and relied solely on internal funds and in-kind commitments (i.e., personnel and resources) to achieve study aims, and had no fixed sample size. Recruitment took place each year at two large annual rural events that each attracted an estimated 50,000–100,00 visitors per event, with researchers attending 3–4 days per event. A total of 674 individuals across seven large rural events were recruited (mean ± standard deviation, 96 ± 42). This study was halted when the large-scale events were cancelled, due to the COVID-19 pandemic. Recruiting was again tried when the events were restarted, with modifications to the intervention to reduce contact between researchers and participants. This had poor recruiting outcomes, and the decision was made to put the study on hold until further notice.

Recruitment for Health SMaRT took place over one year (2018) across two rural markets, through a rural charity, one large rural event and promotion through local networks. Although the project completed 72% of the interviews required, it conducted more health checks than planned *n* = 66 (target *n* = 40–50). Also, some proposed interviews were adapted to interviewer administered surveys, as no follow-up interviews from people attending rural markets were achieved, despite seven people giving permission and details on the day (see Table 2). This study did not use all the allocated external funding, as a PhD candidate was added to the project, and they performed an analysis the data, which had been costed as an external task. In-kind support was required for branded stall equipment and participant resources.

### Personnel and sample size

**Fig. 1:**
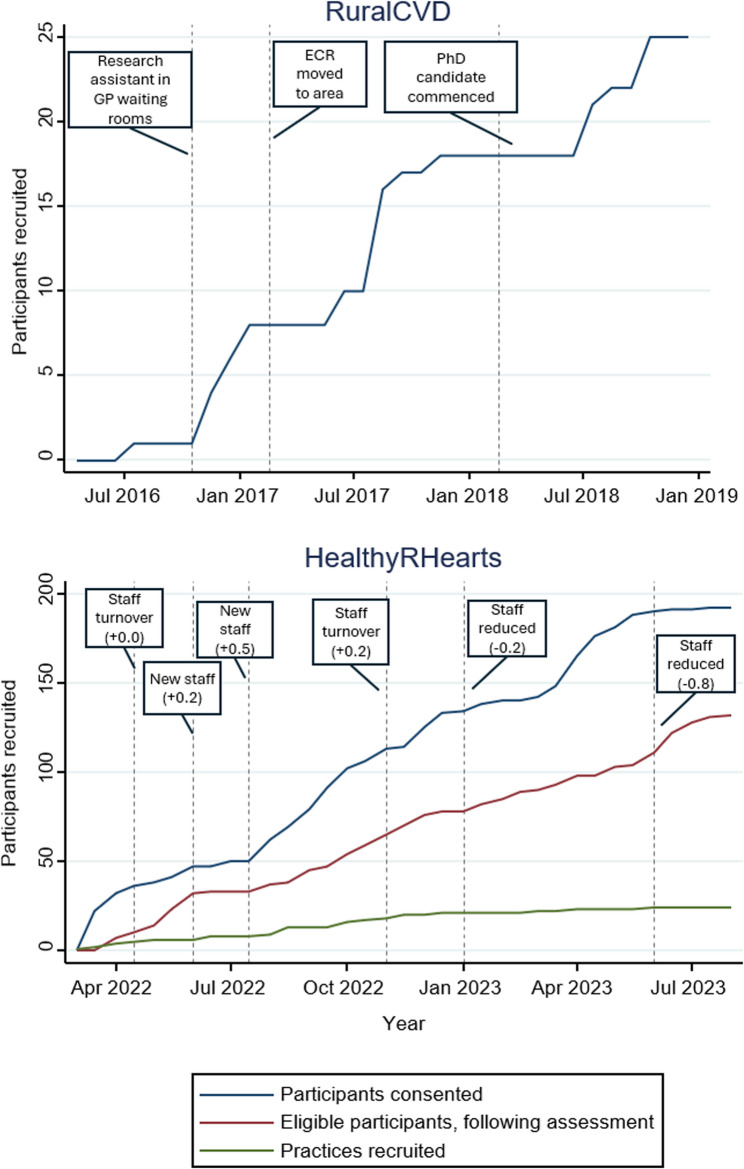
The effect of personnel over time, using trusted local intermediaries

The impact of personnel, used in conjunction with TLI is presented in Fig. 1. RuralCVD employed a second RA, specifically to proactively assist TLI recruitment for a relatively short period of time, and it had immediate effects on participants recruited, increasing from one participant to eight over three months. Also noticeable were the effects of a local ECR, then the PhD candidate on recruitment, although these took longer to take effect. Similarly, in HealthyRHearts, staff turnover and the addition of new staff with a larger FTE also had noticeable effects on participants recruitment. Changes in FTE had less noticeable effects after staff had held the role for longer periods.

**Fig. 2:**
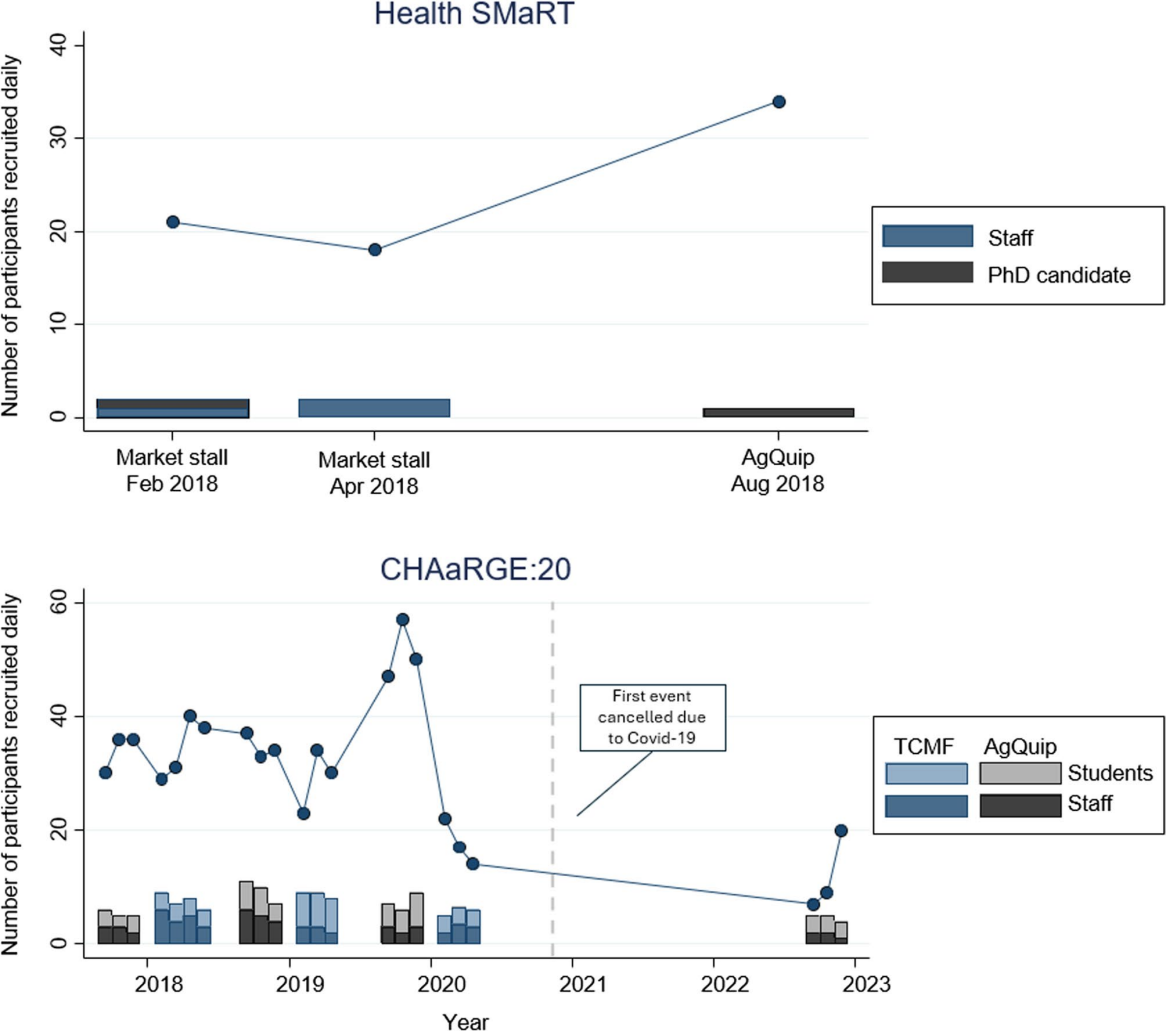
The effect of personnel over time, with researchers performing their own face-to-face recruiting

Relationships between staff and the number of participants recruited using the f2f method are more difficult to evaluate (see Fig. 2). CHAaRGE:20 shows relatively stable recruitment of participants, followed by a large spike at a single festival, prior to the outbreak of COVID-19, followed by a subsequent drop. Changes were made to activities in the post-COVID period, to minimise contact between people, which may have affected interest. For example, at the sole post-COVID CHAaRGE:20 event, participants were only provided the option to complete the activities that could be delivered via survey, such as the eating survey, and weight and waist circumference measures were not offered. Similarly, there is no obvious relationship between staffing levels and participants recruited in Health SMaRT, with the sole PhD candidate recruiting more people at the larger event, albeit over three days, compared to two personnel for a single day at each of the smaller market stalls.

### Summary of TLI proposition data

For studies using TLI as a recruitment method, research capacity building linked to skills and confidence, partnerships and collaborations, and continuity and sustainability were all heavily impacted by staff turnover during the research periods (see Supplementary Table 1). RuralCVD had four staff members take on the recruiting role over three years. HealthyRHearts had eight people undertake the recruiting officer role at varying FTEs over two years. Turnover of practice staff was ‘close to practice’ and impacted partners capacity building. Of note is that a quarter (23%) of the practices who declined to participate, did so because of staff shortages or turnovers. This contributed to unexpected time delays in HealthyRHearts, where the length of time required for practices to attend to tasks related to the research project was severely underestimated and needed to incorporate the unanticipated impact of COVID-19. In relation to infrastructure, accounting for preferences in how secure data transmission was achieved was a predominant challenge in both studies, requiring multiple programs and services to be set up to accommodate difference in individual practices. Additionally, pathology services were easily procured when the recruitment size was limited to single town, but required contracts with more than one major service provider when the project covered a large geographic area, as no one service provided coverage across the entire region.

### Summary of f2f proposition data

When the studies used f2f as the recruitment method, research capacity was very closely linked to resources that were already available, and the studies were more simply designed (see Supplementary Table 2). Both required significant pre-existing infrastructure. Changes to the studies were based on recruitment outcomes and to make the interventions closer to practice. Both interventions actively performed activities that aimed to find out information that was closer to the practice of the participants. Activities were also modified or changed to better suit the environments in which they were delivered. For example, in CHAaRGE:20 the dietary survey took longer than expected, therefore a briefer version was used and participants who were interested, offered the full online version to be completed in their own time. Most noticeably, asking participants to engage with the researchers after the event was poorly responded to. Whilst consent forms for future interviews were provided to approximately 15 people, none were returned.

Confidence and skills developments was important in both studies. In CHAaRGE: 20, no new staff were required, instead undergraduate students were involved at the large-scale event as a further opportunity to develop skills related to their practical placements. A known-level skill and confidence building was planned for, and they were expected to turnover annually. The research capacity building for the smaller Health SMaRT team was more consistent and aimed for continuity and sustainability.

### Summary of qualitative data in relation to the propositions

All study propositions were inextricably linked to one another in the qualitative data, although ‘place-based’ was a core feature in most of the quotes (see Supplementary Table 3).

When RuralCVD was conducted, the concept of ‘place-based’ research had received limited emphasis in the literature. *“There was this idea back then that you could just take what happened in [regional city] and plunk it here [rural town].” – AI/team member RuralCVD (local).*

Additionally, there was an identified need to better understand the needs of the surrounding rural and remote communities, in order to target their research better, ensuring future research was ‘close to practice’. *“Part of what we were trying to do was to try and find out which diseases or health conditions people actually have. Then use this information so we could target our research a lot better.” – CI CHAaRGE:20 (local).*

It was apparent early on that place-based was important, although, this was difficult when recruiting rural dietitians to conduct the research intervention: *“Initially we only wanted dietitians who were rurally based*,* so they are known to people in the communities. However*,* this was a challenge due to the limited staff pool. Therefore*,* we had to broaden it out for any dietitian who had worked rurally. We also needed a diverse range of dietitians who had lived rural experience and who also were available to deliver the intervention in the evenings to accommodate farmers who often did long days and were often unable to take time out and shift workers.” – CI HealthyRHearts (metro).*

The role of place-based, within skills and ‘close to practice’ were also demonstrated: “*This project was my PhD in a regional city*,* and it worked there. When I moved to [rural town]*,* it took me a year to figure out that what I was doing was not working and I had to unlearn what I learned in [regional city].” – ECR/project manager RuralCVD (local)”.*

Due to the lack of literature, it was difficult to plan time, resources and strategies that were ‘close to practice’: *“One of the challenges was that ‘it takes this long to recruit’*,* but it took longer to recruit*,* and required more resources. When writing the grant*,* the articles used to inform the grant were not just based on rural studies*,* therefore*,* the timelines to recruit were not reflective of actual recruitment in rural primary care practices. When you look at data to inform the grant application*,* there were not enough rural studies*,* so we probably were not as aware that the timeline for achieving our sample size was going to be longer.” – CI HealthyRHearts (metro).*

‘Close to practice’ was interwoven with partnerships, as the relationship between researchers and TLIs faced problems when research processes didn’t match the reality of general practice: *“Trying to catch general practice staff in a quiet moment is very*,* very challenging. … That could at times take weeks. Also*,* at many of these rural practices*,* staff often have multiple roles and sometimes have to take on additional roles to cover for somebody that is not there.” – CI HealthyRHearts (local).*

Continuity and sustainability of research officers in RuralCVD and HealthyRHearts was proven a challenge due to only being able to offer part-time positions: *“A lot of time went into building their skills and confidence up. The main reasons why many left was because they were offered full-time continuous positions elsewhere*,* all I could offer was part-time short-term contracts.” – CI HealthyRHearts (local).*

For the f2f studies, continuity and sustainability meant having a core research team, getting a PhD student onboard or creating a staff pool to help out with the recruitment when needed. *“We tried to bring through new staff each year. Some have stayed whereas some have left. We now have a handful of the people with enough skill that you could call up for help*,* if need be.” – CI CHAaRGE:20 (local).*

## Discussion

The aim of this case study was to explore two different participant recruitment methods used by a place-based Australian rural research team, across four separate studies between 2016 and 2024. In particular, this study aimed to explore factors associated with research capacity, and to determine their effect on recruitment timeframes and participant recruitment outcomes. Studies using general practices as their TLI took considerably longer to recruit participants than was planned initially, and neither study using TLI achieved their a priori sample size. While larger sample sizes were achieved when researchers performed their own f2f recruiting, this method required attendance at multiple events over extended periods of time, entailing the transportation of personnel and resources to the various locations. The continuity and sustainability of researcher staffing had a major impact on all four studies, as did the building of skills and confidence of research staff and students. Ensuring the research was ‘close to practice’ also underwent an iterative process of adaptation, most noticeably when using TLI, to match the changing needs of the researchers, intermediaries, and participants. Continuity and sustainability of staff, as well as the need for interventions to be close to practice were concepts that emerged as key factors, often overlapping with the other propositions. Adaptations made to ensure research processes had direct relevance to decision making included determining motivations for engaging with research and aligning research processes with the realities of general practice.

The GP practice recruitment rate in HealthyRHearts (21%; 1–7 GPs per practice) was within the range of other studies that have recruited individual GPs (ranged between 4.6% and 37%) [39, 40], although recruitment methods varied between studies. Most similar to HealthyRHearts, Lech and colleagues [39] (who reported a 4.6% recruitment rate) used the total of (i) a randomly selected sample of GP’s chosen from an insurance database, (ii) face-to-face visits with selected GP practices, and (iii) the number of GP’s who responded to advertisement via social media, newsletters and conferences, to calculate the recruitment rate. McCarthy and colleagues [40] (who reported a 37% recruitment rate), based their recruitment rates on the proportion of practices who had expressed an interest and were formally invited to the study.

In HealthyRHearts, more than half of the practices who declined participation had previously showed interest in taking part in the study. The most common reason for decline was ‘lack of time’ and ‘staff changes/turnover’. ‘Lack of time’ for participating was a consistent finding in the literature for studies who had recruited primary care GP’s [40, 41]. Declining to participate in HealthyRHearts was also likely have to been influenced by the onset of the COVID-19 pandemic, a time when the primary care sector was under pressure, and staff turnover, among other issues, was exceptionally high [42, 43]. Indeed, many research studies affected by the COVID-19 pandemic, regardless of location, experienced considerable challenges with recruitment.

However, the relevance of the research topic to the focus of the GPs and their practice may have also been a factor whether to prioritise time to join the study. Mismatches between the aims of the researchers and the interests of rural health professionals have been described by others [44]. Whilst the recruiting areas described here have a known high rate of cardiovascular disease [37], analysis of referrals to dietitians in the Australian context found that most referrals from registrars (trainees in general practice) were for overweight/obesity or type 2 diabetes [45]. GPs have also reported a mixed experience when referring their patients to a dietitian, ranging from not available in some areas, challenges with referrals, lack of communication from the dietetic service and stigmas surrounding weight of the dietitian and how that was perceived to relate to the service they provide [46]. However, dietitians have reported to feel they are able to provide a more effective service when they are an integrated part of a multidisciplinary general practice, have sufficient experience and are provided with flexible working service delivery contracts [47].

A benefit of recruiting using f2f is the ability to talk to recipients directly at the time of the intervention. While the time and effort in attending and setting up the Health SMaRT stalls in the small market did not result in large recruitment numbers, the research team reflected on the perceived value felt by the attendees in having the researchers present in their rural community, offering services related to health. Recruitment strategies are often labelled as successful or unsuccessful based on the number of people recruited. This notion may need to be re-evaluated for recruitment of people in rural and remote areas, as mistrust has been reported as a common barrier in recruiting rural people to research [10], and physical presence of researchers at local events can help reshape this attribute. Current research on consumer engagement describes the benefits of engaging community members in research that affects them, specifically aiding in the building of trust [48]. Despite being considered time consuming, using large events as a f2f recruitment strategy may add to the limited literature to date on useful strategies when recruiting for rural and remote health-related research.

A notable finding from the f2f recruiting was the lack of response to follow-up telephone interviews in the Health SMaRT study. Whilst half of the sample indicated they would be happy to participate in an interview at a later time, no interviews were achieved. This is not dissimilar from other Australian research, where people were invited to an in-person event from a telephone call about public health and participation in research, with 167 expressing interest, and two attending [49]. A large study covering 12 major cities in the United States seeking to understand drug use in the last 12 months, used a f2f method to recruit 3,045 people anonymously to a survey, with 13.6% also providing a hair sample. Sex, race, and the number and type of drugs used in the last 12 months impacted on whether a sample was provided at the time [50]. These recruitment examples may indicate that there is a point where the recruiting into research being performed, is no longer close to practice for participants. Further, recruiting f2f compared to using TLI’s may be considered more convenient and timelier as the interaction (including any assessments) can be conducted on the spot. As discussed earlier, it was very challenging to engage those recruited during the events for interviews post the event suggesting that it f2f recruitment may not be the most effective strategy when requiring ongoing engagement. Therefore, more consumer engagement needs to be done to understand the stage at which potential participants may no longer engage. All included studies, except Health SMaRT, spent a considerable amount of time on upskilling personnel to ensure recruitment was conducted as per protocol. While Health SMaRT included upskilling, it was mostly included as part of the supervision of the PhD candidate. Several factors including high turnover of staff and adaptations made during the study periods, resulted in unanticipated additional time taken to upskill personnel. The nature of grant funded research positions made continuity and sustainability of staff challenging, due to some staff being offered more secure employment elsewhere.

The sustainability of health research in rural areas is known to be challenging, due to the inconsistent nature of research funding [16, 17, 44]. In addition, due to the limited staff pool in rural and remote settings, this case study demonstrated challenges with recruiting personnel with the right qualifications and entry skills needed for the positions. This often resulted in additional time spent upskilling. Given the additional challenges with employing and retaining research personnel and the additional time taken to recruit participants, it is recommended that future rural-based studies consider additional costs for staffing and time allocated for upskilling, when conducting research in rural and remote areas.

### Strengths and limitations (limitations based on scarcity)

This study adds to the limited literature of recruiting rural participants to research using a place-based team. Additionally, very little evidence is published regarding the evidence of ‘close to practice’ health research in rural area. Cross-case synthesis with pattern matching using four individual studies (cases) embedded with multiple units of analysis allowed for a comparative study design. Studies were performed before, during and after the COVID-19 pandemic, and the effects on the findings of this is unknown. Additionally, it is acknowledged that the data for this case study were derived from a single rural area in NSW, Australia, and therefore due to this single context, may not be able to be generalised to other areas. Further research should be undertaken to compare these findings with other rural areas in both Australia and internationally.

### Implications for practice

This case study highlights the importance for undertaking preliminary work in rural communities where studies will be conducted. Research outcomes were more likely to be achieved when the project aligned with the immediate usefulness and relevance to the populations involved. This may take the form of understanding participant burden or relevant organisational structures, connecting groups and businesses or allocating remuneration [51]. Proactive recruitment, such as using the f2f method, may provide benefits such as researchers being seen as active members of the community and therefore assist in breaking down barriers due to lack of trust. As such, the notion of ‘recruitment success’ in rural communities may need to be reevaluated to focus more on factors related to engagement in research rather than the number of people recruited. Whilst place-based researchers are best positioned to understand their local population, an extensive workforce may not be available, or able to be seconded from other roles. Additional financial support and training may be required to secure appropriately trained staff for research positions involved in participant recruitment. Further, this case study emphasised that additional time and funding may be required if TLIs are used for recruitment in rural settings. Processes undertaken must be carefully aligned with existing practice, while taking into account the demands placed on general practices in these areas.

## Conclusion

Recruitment in rural settings is complex with many factors impacting recruitment success, including continuity and sustainability of research personnel and research being ‘close to practice’. Greater emphasis must be made to more clearly describe recruitment methodology, especially in studies targeting underrepresented populations. Additionally, more clarity is needed from existing studies to accurately budget for the range of challenges encountered in rural settings. A systematic review investigating these factors would benefit the planning of future studies for these populations. Upcoming studies should also develop strategies to minimise disruptions in continuity, and ensure the research focus and processes are relevant to participants, local research team, and TLIs if they are involved.

## Supplementary Information

Supplementary Material 1.

Supplementary Material 2.

Supplementary Material 3.

Supplementary Material 4.

Supplementary Material 5.

## Data Availability

Data relating to the case study may be available from either of the Chief Investigators in the author list.

## Abbreviations

ACTRN: Australian Clinical Trials Registration Number (Australian New Zealand Clinical Trials Registry)
ASR: Age standardised rate
CVD: Cardiovascular disease
E/MCR: Early/Mid-career researcher
F2f: Face to face
FTE: Full time equivalent
GP: General Practitioner
HealthyRHearts: Healthy Rural Hearts (research project)
n/a: Not applicable
NW NSW: Northwest New South Wales (Australia)
PhD: Doctorate of Philosophy
RA: Research assistant
RCT: Randomised Controlled Trial
TLI: Trusted Local Intermediary
UONDRH: University of Newcastle Department of Rural Health
