# Supplementary material for: Exploring recruitment strategies for place-based research in rural areas of Australia: a comparative case study analysis
Source: BMC Prim Care. 2025 Nov 26;26:379. doi: 10.1186/s12875-025-03055-x (PMC12659055; doi:10.1186/s12875-025-03055-x)
Supplement: Supplementary file 1 — Supplementary Material 1. [file 12875_2025_3055_MOESM1_ESM.docx]

Supplementary Materials 1: Interview guide

| **Principle (propositions)** | **Questions** |
| --- | --- |
|  | Clarify role for each study |
| **Research capacity is built by developing appropriate skills and confidence:** | To upskill people within the research team, what did that look like in your role?  ***Possible prompts:***   - - Who was the training for?   - How long did it take to prepare for and provide training/workshop?   - Were there any additional material/resources developed related to upskilling?   - Compared to what was initially planned, did you have to do anything extra to ensure staff had appropriate skills and confidence to perform task?   Post upskilling research team, did you provide any supervision/mentoring?  ***Possible prompts:***   - What did that look like? - How long did that take? - Who was it for?   Were there any challenges with upskilling the research team?  ***Possible prompts:***   - For the TLI trials specifically, what was your role in developing/ providing training for staff at GP practices? - Compared to what was initially planned, did you have to do anything extra to ensure staff had the appropriate skills and confidence to perform the tasks?   Was there any need to upskill people in GP practices? |
| ***Research capacity building should include elements of continuity and sustainability*.** | What strategies were included to promote continuity and sustainability of the research staff?  ***Possible prompts:***   - - Were there any challenges in achieving this?   What strategies were used to promote continuity and sustainability of GP practices and their clinical and administrative staff, to support them during recruitment?  ***Possible prompts:***   - - Were there some challenges to achieve this? |
| ***Appropriate infrastructures enhance research capacity building*** | For the research team, were there any equipment, spaces and technologies that were required to be purchased for each study?  ***Possible prompts:***   - - Where any of these planned or unplanned?   For GP practices, were there any equipment, spaces and technologies that were required to be purchased for each study?  ***Possible prompts:***   - - Where any of these planned or unplanned? |
| **Research capacity building should support research ‘close to practice’:** | For each project, what was your previous experience with recruiting for a CVD trial in a rural context?  In your opinion, what strategies/processes were planned to ensure the research was relevant to the community?  ***Possible prompts:***   - Did any changes occur during the trial?   For recruitment specifically, what do you think was the most important factor that influenced recruitment success? |
| **Linkages, partnerships and collaborations enhance research capacity building** | What strategies were used to develop and support partners (and /or their staff)?  ***Possible prompts:***   - Were there any challenges in maintaining the partnerships?   Were partnerships/linkages or collaborations created with any community bodies to support the f2f trials?  ***Possible prompts:***   - If yes, how was this done and what strategies were used? |
| **Other:** | Is there any else you would like to add that hasn’t been discussed in the questions above? |
