# Supplementary material for: Exploring recruitment strategies for place-based research in rural areas of Australia: a comparative case study analysis
Source: BMC Prim Care. 2025 Nov 26;26:379. doi: 10.1186/s12875-025-03055-x (PMC12659055; doi:10.1186/s12875-025-03055-x)
Supplement: Supplementary file 2 — Supplementary Material 2. [file 12875_2025_3055_MOESM2_ESM.docx]

| **Supplementary table 1**: *Planned strategies, outcomes and adaptations, based on propositions affecting the internal research team, or others external to the team for the TLI trials* | | | |
| --- | --- | --- | --- |
| **Study**  **(recruiting size)** | **Planned for recruitment** | **Outcomes and adaptations** | |
| **Principle (proposition):** *Research capacity is built by developing appropriate skills and confidence:* | | | |
| RuralCVD  (small recruiting area) | **Internal:** Research personnel: on the job learning, with mentoring available by more senior research staff.  **External**: Primary care teams: No upskilling planned. | High turnover of research staff in charge of supporting practices (four different staff at 0.2 FTE over 3 years). CI’s required train new research officers.  *Unforeseen need for:*   - On the job learning for the research team, with additional research skills required at most levels. | |
| HealthyRHearts  (large recruiting area) | **Internal:** Research recruiting officers: 1 x 2-day workshop + training manual, templates, “How to” guides, and sample packs. Active supervision and mentoring by project manager for 2-3 months.  **External**: Primary care teams: Pre-written letters of invitation appropriate for mail merge, guidelines and instructions, “practice packs” containing information about the study and referral to external partners for practice-based information/assistance. | High turnover of recruiting officers in charge of supporting practices (i.e., eight individual research officers at varying FTE over 2 years). Project manager required to train new research officers. Additional FTE required at key recruiting times for short periods.  *Unforeseen need for*:   - Additional assistance. 20 (17-27) correspondence contacts (emails/phone) with practices post receiving practice consent. - Support materials (e.g., YouTube videos) for practices to conduct processes for recruitment. | |
| **Principle (proposition):** *Research capacity building should support research ‘close to practice’* | | | |
| RuralCVD  (small recruiting area) | **Internal:** Staff at 0.2 FTE to recruit/support GP practices.  **External**: Participants were recruited through study flyers at GP practice Participants identified by GP and provided with an information sheet and consent form.  GPs to complete the screening information form required by the researchers.  Consenting participants to be contacted by the researchers via email, with a link to complete surveys and provide information for stage 2 (i.e., consultation with dietitian and follow-up blood test). | The 0.2 FTE position included other tasks than only supporting GP practices.  Ethics variations submitted related to project (n=3) and staffing changes (n=4)  *Unforeseen need for:*   - Online participant consent - Recruitment material to be displayed in GP waiting room. - Physical handouts for patients can give to their GP to check for eligibility. - Presence of a 1.0-FTE RA at the GP practice for three weeks to answer any questions; and to allow the RA to collect consent using an iPad at practice. - Interested people able to leave their contact details with reception to have the consent and information statement sent to them. - Reminders to those not accessing the information statement following their cholesterol test. | |
| HealthyRHearts  (large recruiting area) | **Internal:** Recruitment officers to have AQF 6+ in nutrition, medicine or allied health, previous experience with primary care and currently living in a rural community.  **External**: GP practices within the Hunter New England and Central Coast Primary Health Network footprint, located in Modified Monash Model level 3-6 areas and with a PENCAT agreement with local PHN.  Contact made via practice manager or equivalent and provided with a ‘Practice pack’, containing relevant study material for consideration. Recruiting officer booked onsite visit when possible.  Participants selected for invitation based on risk factors listed in practice software and assessed for eligibility based on heart health service item with rebate available through Medicare.  Practices to receive $100 payment for each participant randomised to the study as administrative compensation.  Health data collected by the study is made available to participant’s GP. | Due to the limited staff pool, one person recruited for the recruitment officer role had no health qualifications, although was experienced in an administrative role.  Of n=126 possible practices, 7% (n=9) were no longer operating, 19% (n=24) initially provided practice consent and 74% (n=93) declined to participate.  Of the practices who declined to participate, n=62 (66%) indicated that they were interested in the study. Main reasons for declining were lack of time n=24 (26%) and staff turnover/ shortages n=21 (23%), with n=41 (44%) not providing a reason.  *Unforeseen need for:*   - Practices to account for COVID-19 and its associated health risks in local areas - Generic invitation for GPs to issue at reception to patients coming in for other medical concerns. - Full study documentation required online, in addition to emailed and hard copies for both General Practices and participants. - Referral to partner organisation for assistance in electronic referrals using current technology. - Posters and Facebook posts for GPs to use on their social media.   *Unexpected time delays:*   - Time between initial contact and:   - receiving practice consent: 32 (13-63) days, with 5 (4-8) correspondences.  - declining practice consent: 98 (16-180) days, with 4 (2-7) correspondences.   - Time to obtain GP consent: 58% of practice provided their first GP consent simultaneously with providing practice consent. The remaining 42% of practices required 39 (15-60) days to provide GP consent. - Time between practice consent and first recruiting letters sent: 83 (38-139) days. - Project ethics variations submitted (n=12) and staffing changes (n=10). | |
| **Principle (proposition):** *Linkages, partnerships and collaborations enhance research capacity building* | | | |
| RuralCVD  (small recruiting area) | **Internal:** The appointed research staff member to find and practice champion develop a working relationship.  **External**: Single practice only targeted for partnership. | Recruited a RA for 1.0 FTE to support recruitment at the first GP practice for 3 weeks. The 0.2 FTE position included other tasks unrelated to recruitment.  Two more GP practices were recruited to boost low participant rates (Total: n=3 GP practices). | |
| HealthyRHearts  (large recruiting area) | **Internal:** Rural CIs and project manager to use local networks to advertise study.  Recruiting officer designated to be consistent point of contact for individual practice, staff and their participants for continuity of care.  **External**: Invite all GP practices within the New England and North-West area of New South Wales. | The recruiting officer role was split, for different officers to focus solely on practices and their staff or to participants.  *Unexpected time delays due to need to build relationships:*   - High turnover of recruitment officer (eight different recruitment officers in 2 years). - High reported turnover of administrative staff at the GP practices. | |
| **Principle (proposition):** *Research capacity building should include elements of continuity and sustainability.* | | | |
| RuralCVD  (small recruiting area) | **Internal:** Recruited two previous locally based honours-level postgraduate students to the recruiting role. | Recruited a locally based PhD candidate to the project, also an honours-level postgraduate. ECR moved from major city but had completed extensive undergraduate local placements. | |
| HealthyRHearts  (large recruiting area) | **Internal:** Have one locally based research officer in charge of recruitment and continuity of support.  Re-hiring of staff working on previous short-term research contracts.  **External**: Intervention designed to mimic real-world application in primary care. | Most practices providing practice consent completed the trial (n=19, 80%).  Out of the practices providing consent, a median of 2 GPs (IQR= 1-3, range 1-7)) were recruited per practice.  *Unforeseen need for:*   - Due to high turnover of research officers and practice administrative staff, continuity of support was challenging to provide. | |
| **Principle (proposition):** *Appropriate infrastructures enhance research capacity building* | | | |
| RuralCVD  (small recruiting area) | **Internal:** Foreseen infrastructure included a pathology contract, statistical support, online data capture (Survey Monkey) and online secure cloud-based data storage that allowed for secure data transmission (OwnCloud).  **External**: No additional infrastructure foreseen. | Data transferred to REDCap close to study completion.  *Unexpected time delay*   - Log in details for secure cloud storage infrequently used to upload data, leading to lost log in details, and consequent practice staff frustration at time wastage. | |
| HealthyRHearts  (large recruiting area) | **Internal:** Foreseen infrastructure included pathology contract with single service, online secure data capture and storage (REDCap), secure appointment system for allied health providers (Halaxy), secure telehealth system for intervention delivery (HealthDirect) and secure transmission of participant health data (BPAC Clinical solutions). | *Unforeseen need for:*   - Pathology contracts with three services, and links to one other - Alternative methods of secure transmission of identifiable and de-identified data: Medical Objects, fax, registered post and secure equipment and transport if data provided to recruiting officer. - Referral to partner organisation to assist with referral system. | |
| *Data reported as median (interquartile range), except where specified)  ***Abbreviations:*** | | | |
| AQF: Australian Qualification Framework  FTE: Full time equivalent | | | PHN: Primary Health Network  RA: Research assistant |
