# Supplementary material for: Exploring recruitment strategies for place-based research in rural areas of Australia: a comparative case study analysis
Source: BMC Prim Care. 2025 Nov 26;26:379. doi: 10.1186/s12875-025-03055-x (PMC12659055; doi:10.1186/s12875-025-03055-x)
Supplement: Supplementary file 3 — Supplementary Material 3. [file 12875_2025_3055_MOESM3_ESM.docx]

| **Supplementary Table 2:** *Planned strategies, outcomes and adaptations, based on propositions affecting the internal research team for the f2f trials.* | | |
| --- | --- | --- |
| **Study**  **(recruiting size)** | **Planned for recruitment** | **Outcomes and adaptations** |
| **Principle (proposition):** *Research capacity is built by developing appropriate skills and confidence* | | |
| CHAaRGE:20  (large recruiting area) | Student training each year prior to major events (2017 onwards), 2 major events per year, approx. 2-3 hours upskilling per session by 1-2 CI’s. Students under direct supervision of at least one CI during events. Training included processes, physical measures, use of equipment, communication, and ethical obligations. | Students upskilled by 1-2 CI’s for 2-3 hours before each of the eight events, and had additional time for practicing unsupervised. |
| Health SMaRT  (small & large recruiting area) | Recruiting aimed to be conducted by E/MCRs and mentoring available by senior CIs on project (no upskilling planned). | PhD candidate added, with training and mentorship provided by thesis supervisors. |
| **Principle (proposition):** *Research capacity building should support research ‘close to practice’* | | |
| CHAaRGE:20  (large recruiting area) | *Events:* Potential participants were attendees of AgQuip (3-day agricultural field day event, Gunnedah) and Tamworth Country Music Festival (TCMF) (10-day event), major rural festivals in rural NSW. Both events expected in excess of 50,000 visitors annually.  *Stall location:* Stalls were clearly branded as a University Department of Rural Health.  - AgQuip: large shed, with other health related vendors.  - TCMF: stall in air-conditioned downtown shopping facility, close to events  *Intervention activities*: Intervention was aimed to be complete brief health assessment in 20 minutes. Participants completed an online survey, then were assessed using a range of measures related to health: height, weight, waist circumference, spectrophotometry, arterial stiffness and dietary survey. Participants were provided with brief verbal feedback by a qualified dietitian at completion.  *Promotion:* Social media, media and word-of-mouth from participants. | Ethics variations submitted related to project (n=3) and staffing changes (n=6). Additional questions about access to health services were added to the online survey.  A derivative activity was developed to determine why people chose to engage with the study, to assist recruitment. Three messages were trialled (n= people approached, % positive response):   - Free health check (n=65, 32%) - Help researchers find out more about rural health (n=70, 33%) - Find out about your own health (n=76, 34%)   *Unexpected time delays*   - Dietary survey took longer than expected. A briefer online version (Healthy Eating Questionnaire) was used as a replacement. Interested participants were offered access to full online FFQ to be completed in own time, with individual analysis provided after completion. - Arterial stiffness was removed, as it took longer than expected to achieve the still and quiet environment required for an accurate reading. - Recruitment halted during pandemic (March 2020 – August 2022). - Recruitment very low at the first event after the pandemic (i.e., AgQuip August 2022). |
| Health SMaRT  (small & large recruiting area) | *Events*: Small-town market, with an unclear expected attendance. Recruiters also attended a local food bank, to recruit people who may have been facing food insecurity.  *Stall location*: University Department of Rural Health branded gazebo, at allocated site in market.  *Intervention activities*: Free heart health checks. Participants could choose one of the following three measures: (i) Australian Eating Survey, (ii) Cholesterol blood check or (iii) Arterial stiffness.  Those meeting the inclusion criteria for interview/focus groups were provided additional information statement and consent form, to return with a self-addressed envelope.  *Promotion (focus groups only)*: Word-of-mouth and recommendation. Brochures also distributed via local networks and connections. | Two ethics variations were submitted in relation to the project.  N=31 participants were recruited from the small-town market. Participant choice of measures were:   - Dietary survey: 10% - Cholesterol test: 39% - Arterial stiffness: 52%   Approximately 50% of market sample expressed interest in interview/focus group. No consent forms were returned. Eight interview/focus group participants gained via promotion.  *Intervention activity:* Only cholesterol checks were performed at AgQuip, during which one-on-one surveys were administered. This was to overcome the challenge of interviewing people later. Only this measure was suitable, as arterial stiffness required a still and quiet environment, and it was impractical for participants to complete an online FFQ, whilst also completing the survey. |
| **Principle (proposition):** *Linkages, partnerships and collaborations enhance research capacity building* | | |
| CHAaRGE:20  (large recruiting area) | A previous partnership existed with an NGO, where medical students assisted in providing health checks at the field day event. The new research activity allowed for allied health to provide a similar role. Health resources (e.g. pamphlets) were also provided from a national NGO, where a partnership also existed with their local representative. | Project was expanded to include two other universities with a focus on rural health and access to similar large-scale events. This did not progress, due to Covid-19. |
| Health SMaRT  (small & large recruiting area) | Health resources (e.g. pamphlets) were provided from national NGO, with where a partnership previously existed with their local representative. | No changes made to linkages, partnerships or collaborations. |
| **Principle (proposition):** *Research capacity building should include elements of continuity and sustainability*. | | |
| CHAaRGE:20  (large recruiting area) | At least one of the two CIs were present at each event. Where possible, the same clinical staff were also part of the investigative team and collected data at least one day per annual event. Students who had opted for a year-long placement were trained, in addition to short-term students on an applicable placement at the time. | Any previous students still able to attend the events were able to return the following year to continue to build skills, if they wished. |
| Health SMaRT  (small & large recruiting area) | It was initially intended that the ECR on the project would most of the recruiting. It was envisaged that a PhD candidate would become part of the team. | PhD candidate took over many of the roles associated with recruiting, as part of their PhD thesis tasks. |
| **Principle (proposition):** *Appropriate infrastructures enhance research capacity building* | | |
| CHAaRGE:20  (large recruiting area) | The intervention was designed to use infrastructure already available to the team. For example: spectrophotometer, weight scales, stadiometer, iPads, tape measures and vehicles to transport personnel and equipment to events.  Health data was originally collected in a paper-based form, as well as SurveyMonkey for online survey (data capture). Feedback was provided on a pre-printed form, and individual values were documented. | All health and survey data (collection and storage) were swapped to REDCap. Participants retained their paper-based feedback form to take away with them. |
| Health SMaRT  (small & large recruiting area) | The intervention was designed to use infrastructure already available to the team or planned within the grant application. For example, sphygmocor for arterial stiffness, cardiochek, an on-the-spot cholesterol test kit and an online FFQ. University vehicles to transport personnel and equipment to events were used, as well as university-branded gazebo for the stall.  Data capture and storage with REDCap was planned. | No changes to infrastructure. |
| CI: Chief investigators  FFQ: Food Frequency Questionnaire (Australian Eating Survey)  NGO: Non-government organisation | | |
