# Supplementary figures and images for: Exploring recruitment strategies for place-based research in rural areas of Australia: a comparative case study analysis
Source: BMC Prim Care. 2025 Nov 26;26:379. doi: 10.1186/s12875-025-03055-x (PMC12659055; doi:10.1186/s12875-025-03055-x)

Supplementary Figure 1: Overview of case study design

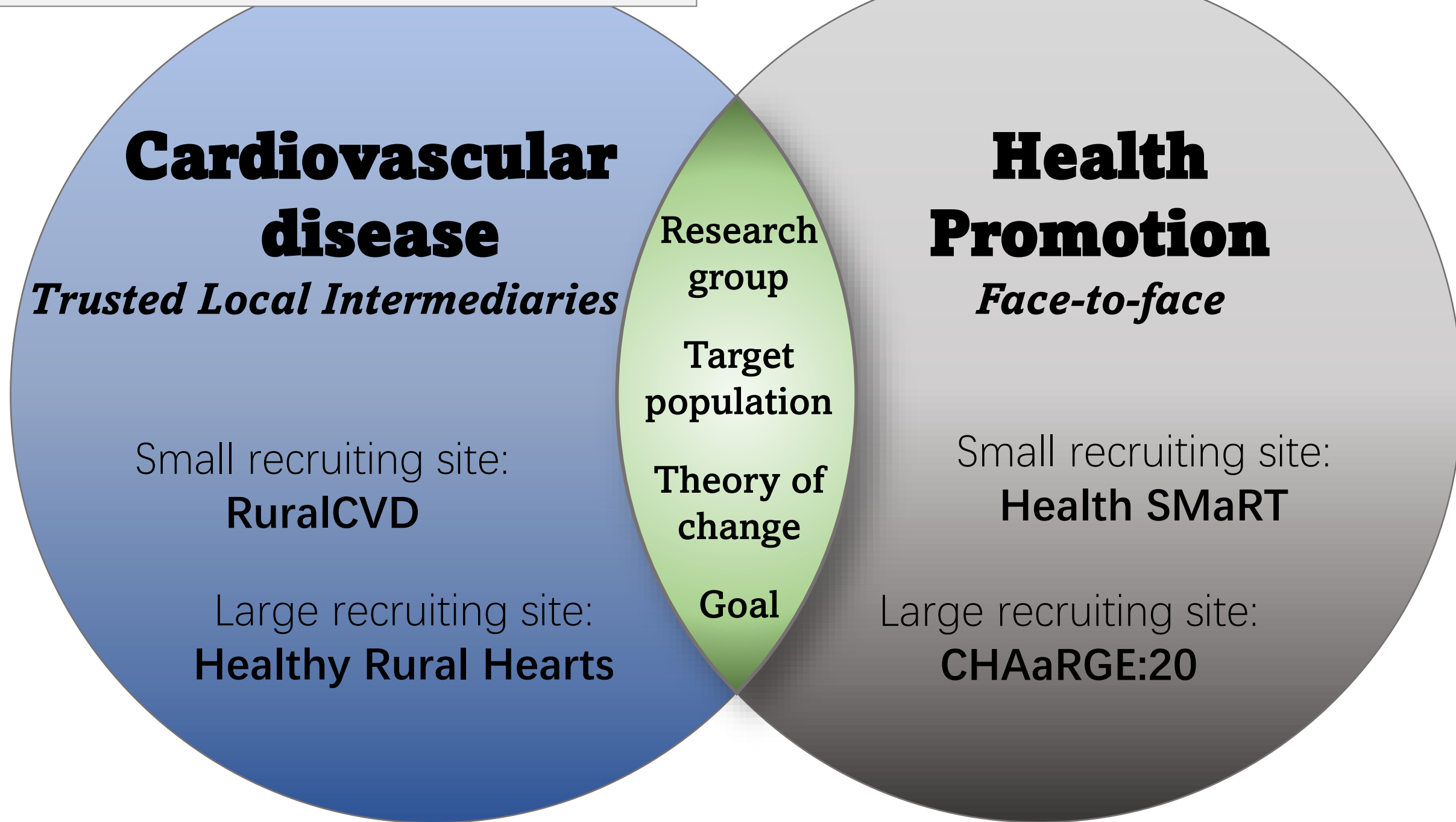

Supplement: Supplementary file 4 — Supplementary Material 4. [file 12875_2025_3055_MOESM4_ESM.pdf]
