# Supplementary material for: Exploring recruitment strategies for place-based research in rural areas of Australia: a comparative case study analysis
Source: BMC Prim Care. 2025 Nov 26;26:379. doi: 10.1186/s12875-025-03055-x (PMC12659055; doi:10.1186/s12875-025-03055-x)
Supplement: Supplementary file 5 — Supplementary Material 5. [file 12875_2025_3055_MOESM5_ESM.docx]

| **Table 5:** *Qualitative comments from chief investigators and research team members* | | |
| --- | --- | --- |
| Principle (propositions) | Study | Qualitative comments relating to the propositions |
| Research capacity is built by developing appropriate skills and confidence: | HealthyRHearts | “The training package took a couple of weeks to prepare, not working on it full time. The actual training session took around a day, and then the observations took probably up to 3 months, per research officer. The observation period was really intense as I had to make sure that all processes were done according to the training, to meet ethics, safety and Medicare requirements.” – CI (local)  “Because I had such a turnover of research officers, I literally lost a year. So a year was spent overseeing and managing staff, and not so many of the other tasks got done because it was all spent on upskilling the staff. The newness of the people coming into the role who didn't have all of the skills that were needed, that was substantial part of my role that I didn't see coming and probably should have. I had to spend a lot more time on upskilling than I had originally planned for.” – CI (local)  “What we assumed were processes that practice staff used regularly, turned out not to be not so, and definitely not in all businesses. We had to develop training for admin staff at the general practices on the processes that were required for the study. That also required upskilling of the research officers, who had not done these things either.” – CI (local) |
|  | RuralCVD | “This study upskilled all of us at the same time, because we were all still learning how best to do our roles were and where we fitted.” – ECR/project manager (local)  “This project was my PhD in a regional city, and it worked there. When I moved to [rural town] I had to unlearn what I learned in [regional city], and it took me a year to figure out that what I was doing was not working.” – ECR/project manager (local)  “I was mostly involved in the early stages of the study, mainly to support and upskill the junior RA with writing the ethics application as she had never done ethics before. I remember having meetings with the CI (located in a regional area) and junior RA and then trying to translate the discussions into what the ethics submission would look like, with lots of changes along the way. It was a learning experience for me as I was not that long out of my PhD.” – AI/team member (local) |
|  | CHAaRGE20 | “The only unexpected things would have been preparing the students to some of those unexpected interactions with people at the events. We [two local CI’s] really had to be with the students at the events as there may be questions participant would raise that the students weren't sure about, particularly if there were mental health issues. Part of the study was to provide participants with a brief nutritional advice or engagement about how they're going, but it always often went beyond that. So there were aspects of the study that were beyond the skill set of the students. But they got to observe us perhaps go in and deal with the crisis. That said, we did actually think that could happen, and part of their training was to get us over when faced with a complex situation.” – CI (local - 1)  “At least for the staff if they had learned it once, then they may have just needed a refresher. Upskilling the students to help with recruitment was a huge part of CHAaRGE each year. Sometimes you would train students up at the beginning of the year for TCMF, and then you might have the same students later in August for AgQuip, but you would typically have some additional new students as well that needed upskilling.” – CI (local - 2) |
|  | Health SMaRT | “We had a small team of only myself (CI local), a PhD candidate and another CI (local). So the majority of upskilling to benefit the PhD candidate.” – CI (local)  “The upskilling had been more layered across a long period of time as Health SMaRT came about after RuralCVD, and I had done other RA work in between. I was there from the start and helping out with the ethics, so I found that really helped to understand what was going on. It also meant that I was able to focus more on other practical upskilling e.g., using the sphygmocor.” – PhD candidate |
| Research capacity building should support research ‘close to practice’: | HealthyRHearts | “We thought we predicted the challenges with recruitment, but we didn’t. Then the COVID-19 pandemic occurred, and the challenges became insurmountable within the planned timeframe and budget.” - CI (metro)  “One of the challenges was that ‘it takes this long to recruit’, but it took longer to recruit, and required more resources. When writing the grant, the articles used to inform the grant were not just based on rural studies, therefore, the timelines to recruit were not reflective of actual recruitment in rural primary care practices. When you look at data to inform the grant application, there were not enough rural studies, so we probably were not as aware that the timeline for achieving our sample size was going to be longer.” – CI (metro)  “So one of the challenges is to find a body of research that focuses on recruitment from rural primary practices. If you can classify whether studies in the MM [rurality] categories, think it will allow people to justify their budgets better and have realistic guidelines. Also, if NSW Regional Health Partners could work with the Primary Health Network, and with the Department of Rural Health (for our region) to come up with a consensus document around recruitment of regional documents, that would really help with budgets.” – CI (metro)  “Our community had received some bad press about how unhealthy our town was in this region, and it was just awful to read. It was high up on our agenda to stop people talking about us [our community] that way.” – CI (local)  “What we got wrong in our preliminary work was the close to practice. What we had then was close to practice metro, not close to practice rural. By the time we got to HealthyRHearts, I may have not known about what to do in all instances but I certainly knew what not to do” – CI (local)  “The factor that was most important for recruitment success of general practices was [our] research team. They were all locals and part of the community. The practices were not talking to someone on the end of the phone who were in a completely different town and who had no idea about their situation, community or what was available. The recruitment team were extremely good at building relationship with the people at the practices.” – CI (local) |
|  | RuralCVD | “There was this idea back then that you could just take what happened in [regional city] and plunk it here [rural town].” – AI/team member (local)  “Back then, at no point did I consider that place-based was a thing. That is a valuable lesson that I have learned that you just cannot do that. This project was my PhD in a regional city, and it worked there. When I moved to [rural town] I had to unlearn what I learned in [regional city], and it took me a year to figure out that what I was doing was not working.” – ECR/project manager (local)  “People who progressed through the study were very motivated to begin with. In parts, recruitment was driven by participants who wanted to take part. For example, we had one participant who was so motivated that he was the reason we got an entire practice on board.” – PhD candidate |
|  | CHAaRGE20 | “The students were able to practice the skills that they had learned on placement and get a chance to talk to members of the public. For us, this was as close to practice you can get in terms of students building their clinical and research skills” – CI (local - 1)  “Part of what we were trying to do was to try and find out which diseases or health conditions people actually have. Then use this information so we could target our research a lot better.” – CI (local - 1)  “We thought, who wouldn't want a free health assessment and advice. Obviously because we were offering something we would get their data and ask them questions, and they would get something of value on the spot. So, I guess that was our way of engaging with the community. It was also up to us to find out what's going on out here, because there is not a lot of information available of this nature that is aimed at helping rural people” – CI (local - 2) |
|  | Health SMaRT | “I think we realised that these small communities really valued that people [us] come out and visit their markets and offices, offering something to the residents that's good for their health. Not everyone was interested, and many people walked by, but I think it could have built into something. It's just that it wouldn't be like AgQuip or Tamworth Country Music festival, where there's lots of people coming through, and you've got a lot more potential to recruit participants. It's a lot of effort for small numbers, but it made me feel as though people in these small rural communities valued that opportunity.” – CI (local - 2)  “It was the strategies we offered that tried to ensure the research was relevant to the community. We offered three strategies that we thought would interest people. One was testing cholesterol using finger prick, the other was the Australian Eating survey and the third was measuring arterial stiffness. We made people choose which one they wanted the most, so that we could get an idea about what was more important to people.” – CI (local - 1) |
| Linkages, partnerships and collaborations enhance research capacity building | HealthyRHearts | “On paper, the team had phenomenal expertise, but everyone was spread out. All dietitians were spread out across the region, one CI was located in Armidale, others in Newcastle and Tamworth. The extra challenge was not even that everyone were rurally based, it was that being ‘rurally based’ did not mean being collocated as well. The primary care practices were also incredibly spread out. Resources to go out and talk to people/take things to do, do site visits, or get IT support/fix something, were all exacerbated by distance.” – CI (metro)  “To support the partnerships, we essentially gave them everything that they needed. If they experienced a problem and we could not fix it, then we put them in touch with someone who could, then followed up with them to ensure it had been resolved. We made ourselves incredibly available.” – CI (local)  “The challenge to maintain the partnerships with the GP practices was only having part-time staff and they often had part-time staff too. Trying to catch general practice staff in a quiet moment is very, very challenging. Essentially, everything took longer as you were often waiting for the person you needed at the practice to be available at a time when we [our recruitment team] were available. That could at times take weeks. Also, at many of these rural practices, staff often have multiple roles and sometimes have to take on additional roles to cover for somebody that is not there.” – CI (local) |
|  | RuralCVD | “Being responsive to the practice was important. Because of our low staffing, when they had issues or questions, they sometimes had to wait a week for an answer. Then it was too late.” – ECR/project manager (local)  “I think the other thing that we definitely understand now that we did not do then, is that these GP practices are very, very busy. They don’t have a lot of time, so it is always going to be hard work even with us on our A game. You also really need someone at the GP practice who cares about the project, have some sort of invested interest and the time to help you.” – AI/team member (local)  “I was only involved towards the end of the study. But I remember that building the personal relationship with the champions in the practices worked quite well.” – PhD candidate |
|  | CHAaRGE20 | “We worked alongside HealthWise. We shared the cost of a shed with them, and they would send people our way and vice versa. They also used our medical students for their activities.” – CI (local - 2) |
|  | Health SMaRT | “We were members of the Tamworth CVD working group at the time and Heart Foundation gave us pamphlets and resources to put in our stall to support what we were doing.” – CI (local - 1)  “We did reach out to other organisations, with the aim to get to the hard-to-reach people. But these organisations were difficult to engage with.” – CI (local - 2) |
| Research capacity building should include elements of continuity and sustainability. | HealthyRHearts | “Initially we only wanted dietitians who were rurally based, so they are known to people in the communities. However, this was a challenge due to the limited staff pool. Therefore, we had to broaden it out for any dietitian who had worked rurally. We also needed a diverse range of dietitians who had lived rural experience and who also was available to deliver the intervention in the evenings to accommodate farmers (who often did long days and were often unable to take time out) and shift workers.” – CI (metro)  “The initial plan was that each staff would take a single practice as their contact, to build the continuity with that practice. This proved to be very challenging with part-time staff as the day the practice needed to talk to that staff member, they were not on shift. I also lost a lot of staff, and during the pandemic especially, many of the practices also lost a lot of staff.” – CI (local)  “To try to promote continuity of the recruitment officers, I built the rapport with them to make sure they felt comfortable asking questions and if they needed anything, were happy to ask for it. For example, if they requested more training or resources to do their job, it was provided to them. A lot of time went into building them up. The main reasons why many left was because they were offered full-time continuous positions elsewhere, all I could offer was part-time short-term contracts. The others left because the short-term contracts suited their family life” – CI (local) |
|  | RuralCVD | “The big continuity was getting a PhD student on it full time, ‘cause that was the continuity that was needed and mattered.” AI/team member (local)  “Even the first project staff were from rural placements and later hired onto the project, so there was some continuity of people. The PhD student had just completed their undergraduate degree before coming onboard the project. While they all were new to the project, they were not necessarily new to the town, which is the best that we could asked for as none of us had the skills back then” – ECR/project manager (local)  “I did sometimes get that impression of like, ‘oh, yeah, at least they are doing their PhD, we want to help her out’. People do sometimes express that sort of sentiment when you're with them, which is quite nice.” – PhD candidate |
|  | CHAaRGE20 | “Continuity is us [the two CI’s] hanging around for a while” – CI (local - 2)  “Generally speaking, if you could use the visiting students at both Tamworth Country Music festival and again at AgQuip, that's the best you could hope for in terms of the students.” – CI (local - 1)  “We tried to bring through new staff each year. Some have stayed whereas some have left. We now have a handful of the people with enough skill that you could call up for help, if need be.” – CI (local - 1) |
|  | Health SMaRT | “That was the initial plan was that I [CI local] would do the bulk of the work because it was my application. Whilst the PhD student was not locked in at that stage, there was a fair chance that they would be. So the continuity was that to build the PhD student into the project.” – CI (local - 1)  “It was probably put together quite well from an ethics point of view from the start. We wanted it to be as flexible as possible, so we included options of where we might be able to take it. For example, we put down quite a few different places we could go and set up stalls.” |
| Appropriate infrastructures enhance research capacity building | HealthyRHearts | “I had to buy a lot of different communication technologies to that was compatible with the systems the general practices used. They have about 4-5 different systems that they use, and each practice was unique. Therefore, we as a team had to become really good at all of those. All of our data storage systems also had to be custom built to meet the ethical standards.” – CI (local) |
|  | RuralCVD | “We didn't need a huge amount of resources for this project and there were not too many barriers in getting the few things that we needed. There were opportunities to get small pockets of money, which then allowed us to cover things like the pathology costs.” – AI/team member (local) |
|  | CHAaRGE20 | “There was a number of opportunities to purchase equipment in the lead up to the study, and we had some of the required equipment already. We purchased a few pieces of equipment with various grants that we got.” – CI (local - 2) |
|  | Health SMaRT | We needed to purchase the cholesterol tests and a sphygmocor. The sphygmocor was very expensive, but I got a university grant to cover it. Apart from that, we had all the support for infrastructure.” – CI (local - 1) |
| Key:  AI = Associate investigator; CI = Chief investigator; ECR = Early career researcher; local = living in the rural community; metro = living in a metropolitan area, MM = Modified Monash, TCMF = Tamworth Country Music Festival | | |
